# The ameliorative effect of silibinin against radiation-induced lung injury: protection of normal tissue without decreasing therapeutic efficacy in lung cancer

**DOI:** 10.1186/s12890-015-0055-6

**Published:** 2015-07-05

**Authors:** Yeonghoon Son, Hae June Lee, Jin Kyung Rho, Soo Young Chung, Chang Geun Lee, Kwangmo Yang, Sung Ho Kim, Minyoung Lee, In Sik Shin, Joong Sun Kim

**Affiliations:** Dongnam Institute of Radiological and Medical Sciences (DIRAMS), Busasn, South Korea; Korea Institute of Radiological and Medical Science (KIRAMS), Seoul, South Korea; Department of Pulmonology and Critical Care Medicine; Asan Institute for Life Sciences, Seoul, South Korea; College of Pharmacy, Kyungpook National University, Daegu, South Korea; College of Veterinary Medicine, Chonnam National University, 500-757, 77 Yongbong-ro, Buk-gu, Gwangju, South Korea

**Keywords:** Silibinin, Irradiation, Lung fibrosis, BALF, Lewis lung cancer, Mice

## Abstract

**Background:**

Silibinin has been known for its role in anti-cancer and radio-protective effect. Radiation therapy for treating lung cancer might lead to late-phase pulmonary inflammation and fibrosis. Thus, this study aimed to investigate the effects of silibinin in radiation-induced lung injury with a mouse model.

**Methods:**

In this study, we examined the ability of silibinin to mitigate lung injury in, and improve survival of, C57BL/6 mice given 13 Gy thoracic irradiation and silibinin treatments orally at 100 mg/kg/day for seven days after irradiation. In addition, Lewis lung cancer (LLC) cells were injected intravenously in C57BL/6 mice to generate lung tumor nodules. Lung tumor-bearing mice were treated with lung radiation therapy at 13 Gy and with silibinin at a dose of 100 mg/day for seven days after irradiation.

**Results:**

Silibinin was shown to increase mouse survival, to ameliorate radiation-induced hemorrhage, inflammation and fibrosis in lung tissue, to reduce the number of inflammatory cells in the bronchoalveolar lavage fluid (BALF) and to reduce inflammatory cell infiltration in the respiratory tract. In LLC tumor injected mice, lung tissue from mice treated with both radiation and silibinin showed no differences compared to lung tissue from mice treated with radiation alone.

**Conclusions:**

Silibinin treatment mitigated the radiation-induced lung injury possibly by reducing inflammation and fibrosis, which might be related with the improved survival rate. Silibinin might be a useful agent for lung cancer patients as a non-toxic complementary approach to alleviate the side effects by thorax irradiation.

## Background

Radiotherapy has been used to treat lung cancer, but because radiation can damage normal tissues, many complications may develop in patients receiving lung irradiation [1]. Radiation-induced pulmonary damage to normal lung tissue can lead to early phase pneumonitis and late phase fibrosis months to years after irradiation, which can affect a patient’s quality of life [2]. An animal model for pulmonary fibrosis has been established using total body irradiation at 15 Gy [3]. Previous studies had shown that fibrosis was not induced in C3H/HeJ and CBA/J mice but was found in C57BL/6 mice during the late phase after thorax irradiation [4,5]. Although radioprotective agents, including soy isoflavone, have been found to mitigate the inflammation and fibrosis induced by irradiation, the mechanism of pulmonary fibrosis induced by thorax irradiation is still unknown; however, inflammation was proposed to involve the radiation-induced fibrosis formation [6].

Silibinin, a standardized extract from the fruits and seeds of milk thistle, is the major active constituent of silymarin and is used clinically and consumed as a dietary supplement for liver disease [7,8]. Silibinin has shown promising and potential anti-tumor efficacy in several cancers including lung cancer models [9-11]. Previous research showed that the oral administration of silibinin produced a profound effect in protecting mice against radiation-induced mortality and preventing DNA damage in vitro [12]. However, the potential protective effect of silibinin against radiation-induced lung fibrosis is unknown.

In this study, we administrated silibinin to irradiated C57BL/6 mice to examine the potential radioprotective effect of silibinin in the normal lung tissue. Changes in the number of inflammatory cells were evaluated in bronchoalveolar lavage fluid (BALF), and the histologic change of lung tissue was investigated to verify the inflammatory response and fibrosis at 80 and 200 days following thorax irradiation.

## Methods

### Animals

Six-week-old female C57BL/6 mice (Central Lab. Animal Inc., Seoul, Korea) were used after one week of quarantine and acclimatization. The animals were maintained in a room at 23 ± 2°C, with a relative humidity of 50 ± 5%, artificial lighting from 08:00–20:00 and 13 ~ 18 air changes per hour. The mice were given a standard laboratory diet and water ad libitum. All experimental procedures were carried out in accordance with the NIH Guidelines for the Care and Use of Laboratory Animals and were conducted following a protocol approved by the Institutional Animal Care and Use Committee of the Dongnam Institute of Radiological and Medical Sciences. The animals were cared for in accordance with the dictates of the National Animal Welfare Law of Korea.

### Radiation and silibinin treatment

Each mouse was anesthetized with tiletamine/zolazepam (Zoletil 50®; Virak Korea; Seoul, Korea) and restrained on a tray. Mice were exposed to whole thorax radiation (13 Gy; dose rate 3.8 Gy/min) using 6 MV high-energy photon rays (ELEKTA, Stockholm, Sweden) with a 1.5 cm bolus on the surface. The radiation dose was prescribed at the midline of body thickness. Dose variation inside the lung was estimated to be within 50 ± 5% of the prescribed dose. Sham-irradiated mice were treated in the same manner but without radiation. A dose of 100 mg/kg silibinin was dissolved in PBS and was treated P.O. for 7 consecutive days after irradiation. The dose and dosage was considered optimal for radioprotection and blood concentration as reported earlier [12,13]. Control mice received the same dose and dosage of vehicle in the same manner. Mice were sacrificed at 80 or 200 days after irradiation.

### Survival measurement

Survival was monitored daily and was reported as the percentage of animals surviving until 200 days after irradiation. Each treatment group consisted of 10 mice. Animals were sacrificed when in a moribund state.

### Bronchoalveolar lavage fluid analysis

At 80 and 200 days after thorax irradiation, mice were anesthetized by intraperitoneal injection of Zoletil, and a tracheostomy was performed. Bronchoalveolar lavage fluid (BALF) was obtained by a recently described method [14]. The total number of inflammatory cells was determined by counting the cells in at least five squares of a hemocytometer after cell viability testing with Trypan blue staining. To determine the differential cell counts, 100 μL of BALF was centrifuged (200 g, 4°C, 10 min) onto slides using a Cytospin (Hanil Science Industrial Co.; Seoul, Korea). The slides were dried, and the cells were fixed and stained with the Diff-Quik® staining reagent (B4132-1A; IMEB Inc.; Deerfield, IL, USA) according to the manufacturer’s instructions. The numbers of macrophages, neutrophils, and lymphocytes were calculated using the percentages obtained multiplied by the total yield. Images of slides were obtained using a digital camera mounted on a microscope (Nikon Eclipse 80i; Nikon Corporation; Tokyo, Japan). Differential count of inflammatory cells in BALF was performed in a double-blind screen by two independent pathologists. The average cellular area of individual macrophages was calculated using Image-Pro Plus image analyzing software (Media Cybernetics; Bethesda, MD, USA). The percentage of multinucleated cells was calculated by dividing the total cell count (approximately 400 cells) by the scored cells [15].

### Histopathology

After BALF samples were obtained, lung tissue was fixed in 10% buffered formalin and embedded in paraplast wax to prepare 4 μm thick tissue sections of lung tissue for hematoxylin-eosin staining. Two tissue sections from four different parts of the lung from each animal were prepared for histological examination. Images of lung sections were obtained using a digital camera mounted on a microscope (Nikon Eclipse 80i; Nikon Corporation; Tokyo, Japan). Quantification was performed using Image-Pro Plus image analyzing software (Media Cybernetics; Bethesda, MD, USA).

Each tissue section was given a score from 0–4 based on the amount of area affected by interstitial inflammation, alveolar wall thickening, peribronchial inflammation and interstitial edema (0 ≤ 10%, 1 = up to 30%, 2 = up to 50%, 3 = up to 70%, 4 ≥ 70%) [16].

Lung fibrosis was evaluated in lung sections stained with Masson’s trichome (n ≥ 4) by examining the amount of collagen fibers [14]. Each lung section was given a score from 0–3 depending on collagen fiber thickness and dispersion. Lung sections were analyzed and photographed (Nikon Eclipse 80i; Nikon Corporation; Tokyo, Japan). The degrees of inflammation and fibrosis in the lung tissue were scored in a double-blind screen by two independent pathologists. In case of fibrosis, we additionally performed quantitative analysis using Image-Pro Plus image analyzing software (Media Cybernetics; Bethesda, MD, USA) [17].

### Establishment of lung tumor model

**Figure 1:**
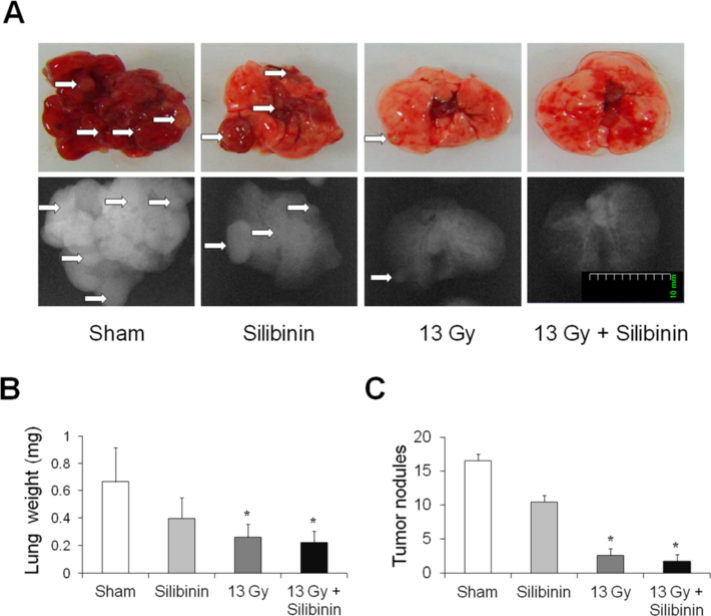
**(A)** Representative photographs and radiographs of mouse lung tissue. Lung weight **(B)** and number of tumor nodules **(C)** at 21 days following irradiation. The data are reported as means ± SEM (*n* = 7 per group). *p < 0.05 *vs.* sham-irradiated controls.

The C57BL/6 mouse Lewis lung cancer (LLC) cells (Korean Cell Line Bank; Seoul, Korea) were cultured in RPMI culture medium containing 7% heat-inactivated fetal bovine serum with supplements. LLC cells, at 1 × 10^6^ cells in 200 ml DPBS, were injected intravenously into the tail vein of seven-week-old female C57BL/6 mice. The mice were randomly divided into the following four groups: vehicle + sham irradiation group (n = 10), silibinin + sham irradiation group, vehicle + 13 Gy irradiation group, and silibinin + 13 Gy irradiation group. Figure 1A shows the timeline for establishment of the lung tumor model. Multiple metastatic lung cancer was established within one week post injection as judged by parallel animals that were sacrificed and evaluated histologically. At this time, the mice received whole thorax irradiation (13 Gy), and 100 mg/kg/day silibinin was administered orally for 7 days after irradiation. Lung weights and lung cancer nodule measurements were determined using a micro-CT scanner (NFR Polaris-G90; NanoFocusRay; Jeonju, Korea).

### Statistical analysis

The data are reported as the mean ± SEM. The data were analyzed using one-way analysis of variance (ANOVA) followed by the Student-Newman-Keuls post hoc test for multiple comparisons. In all cases, a p value < 0.05 was considered significant.

## Results

### Effects of silibinin treatment on the survival rate of irradiated mice

**Figure 2:**
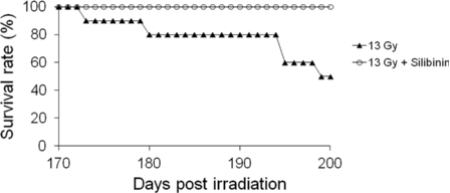
Survival rate of mice treated with radiation alone and radiation plus silibinin. The data are reported as means ± SEM (*n* = 5 per group).

To determine the effect of silibinin treatment after a single dose of radiation, C57BL/6 J mice were exposed to 13 Gy whole-thorax irradiation, and some groups were treated with silibinin after radiation. Although all mice irradiated with 13 Gy survived to 170 days following irradiation, the survival rate of this group dropped to 40% at 200 days after thorax irradiation (Figure 2B). However, all of the mice treated with silibinin after irradiation survived until 200 days, and this survival rate was increased compared with the mice exposed to radiation alone (Figure 2B).

### Effects of silibinin on the level of inflammatory cells in the BALF of irradiated mice

**Figure 3:**
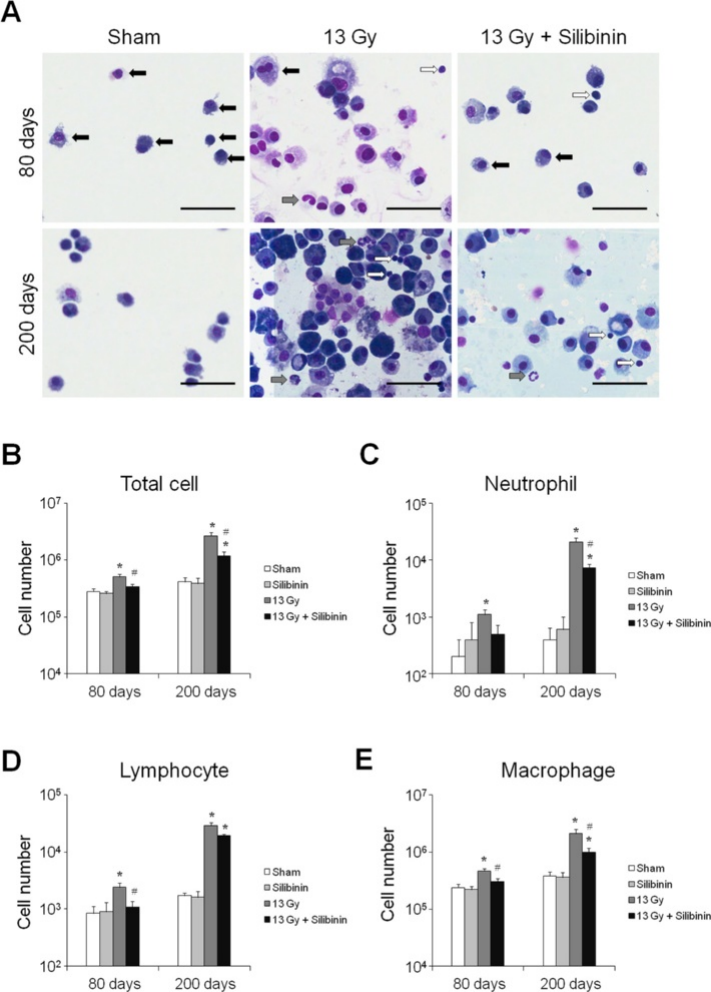
Changes in bronchoalveolar lavage fluid (BALF) of C57BL/6 mice after irradiation. **(A)** Representative pictures of Diff-quick staining of cytospin preparation at 80 and 200 days after thoracic irradiation. Changes in the number of total cells **(B)**, neutrophils **(C)**, lymphocytes **(D)**, and macrophages **(E)** in BALF from C57BL/6 mice. The data are reported as means ± SEM (*n* = 5 per group). *p < 0.05 *vs.* sham-irradiated controls. ^#^ *p* < 0.05 *vs.* irradiated groups.

To evaluate the effect of silibinin on inflammatory cells in the BALF, total cells including neutrophils, lymphocytes, and macrophages were measured after whole-thorax irradiation (Figure 3A). The total number of BALF cells increased significantly at 80 days after irradiation, and the cell number at 200 days increased further. When the cellular component of BALF was evaluated, significant increases were found in each cellular type, both at 80 and 200 days (Figure 3B-E). However, at 80 days after irradiation, the number of total cells, lymphocytes, and macrophages in the BALF of irradiated mice given silibinin were significantly decreased compared with irradiation-only group (Figure 3B-E). Furthermore, at 200 days following thorax irradiation, the number of total cells, neutrophils, and macrophages in the BALF of irradiated mice given silibinin were significantly down-regulated compared with irradiation-only group (Figure 3B-E). These data indicate that silibinin ameliorates the inflammatory response in the BALF of mice following whole-thorax irradiation.

### Changes in macrophage morphology in the BALF of irradiated mice with silibinin treatment

**Figure 4:**
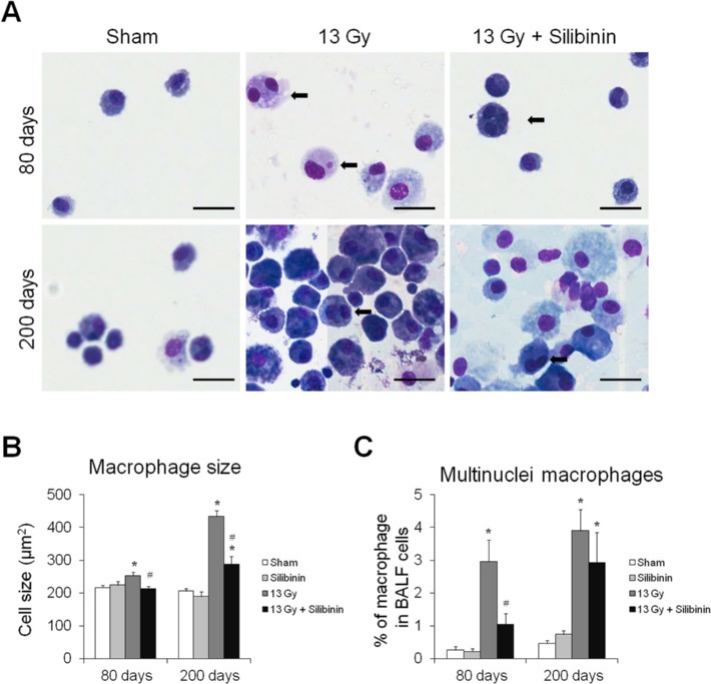
Effects of radiation and silibinin on bronchoalveolar lavage fluid (BALF). **(A)** Representative pictures of Diff-quick staining of cytospin preparation in BALF after thoracic irradiation with and without silibinin treatment. Changes in macrophage size (A) and percentage of multinucleated macrophages in BALF at 80 and 200 days after thoracic irradiation. The data are reported as means ± SEM (*n* = 5 per group). *p < 0.05 *vs.* sham-irradiated controls. ^#^ *p* < 0.05 *vs.* irradiated groups.

Because the main cellular component of BALF is macrophage, macrophage morphology and multinucleated macrophages were evaluated in the BALF of mice after irradiation with and without silibinin treatment. In mice treated with thorax irradiation alone, the macrophage size was increased at 80 days, and enlarged macrophages became notably evident at 200 days after irradiation (Figure 4A and B). After silibinin treatment, the size of macrophages in the BALF was significantly reduced compared to those in the radiation-only group (Figure 4B).

BALF cells from control mice only occasionally contained dividing macrophages. However, mice receiving thoracic irradiation had a high frequency of multinucleated macrophages at 80 and 200 days after irradiation (Figure 4C). A decreased number of multinucleated macrophages was observed in mice treated with silibinin at 80 and 200 days following thoracic irradiation, although the difference at 200 days was not significant.

### Anti-inflammatory effects of silibinin in irradiated normal lung tissue

**Figure 5:**
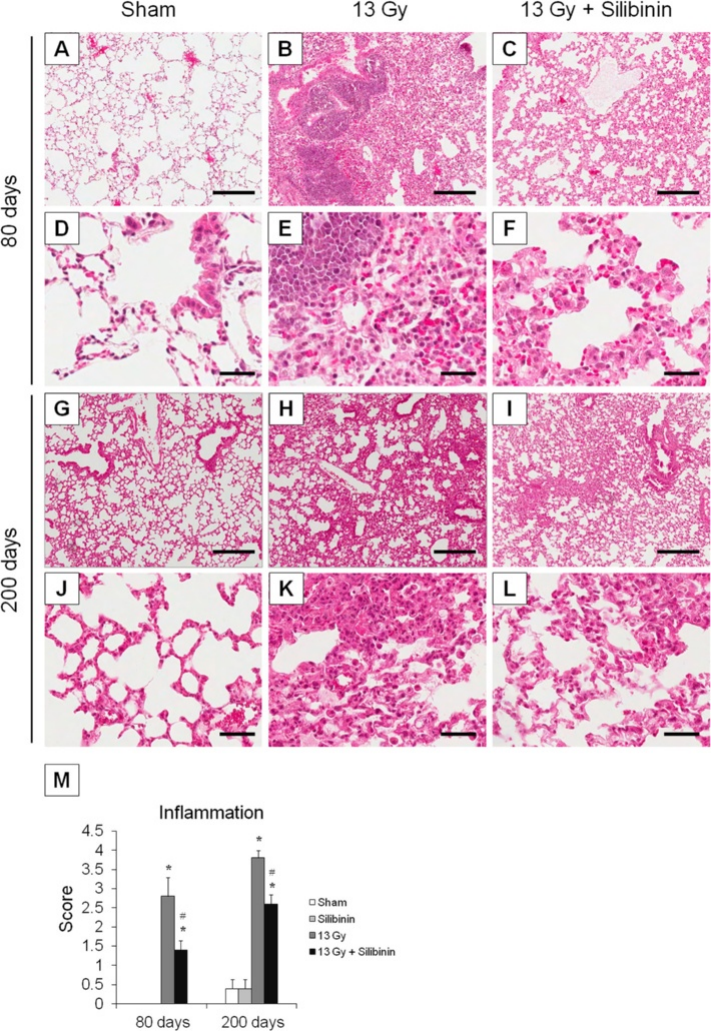
Changes in the histology of lung tissue from mice treated with radiation and silibinin. **(A-F)** 80 days after irradiation; **(G-L)** 200 days after irradiation; **(A, D, G, J)** Lung tissue from sham-irradiated control; **(B, E, H, K)** Lung tissue from 13 Gy-irradiated mice; **(C, F, I, L)** Lung tissue from 13 Gy-irradiated mice with silibinin treatment. Hematoxylin and eosin staining. Scale bars in low magnification present 300 μm. Scale bars in high magnification present 50 μm. (**M**) Inflammation score from lung tissue at 80 and 200 days following irradiation. The data are reported as means ± SEM (*n* = 5 per group). *p < 0.05 *vs.* sham-irradiated controls. ^#^ *p* < 0.05 *vs.* irradiated groups.

To examine the changes in lung tissue after thoracic irradiation combined with silibinin treatment, the extent of inflammation in mice lung was assessed histologically at 80 and 200 days after radiation. In sham control mice, the lung tissue showed normal lung alveoli at 80 days and some areas of thick alveolar septae at 200 days following irradiation (Figure 5A and J). Silibinin treatment alone caused no changes in the morphology of the lung alveoli compared to control mice (data not shown). In the lung tissue of mice that received irradiation alone, infiltration of inflammatory cells was evident at 80 and 200 days after thoraxic irradiation, and extensive hemorrhages were observed (Figure 5B and H). Irradiated mice treated with silibinin in the post-irradiation period showed an ameliorated inflammatory response as well as decreased alveolar septum thickness (Figure 5C and I). When lung tissue inflammation was scored, the increased inflammation score caused by irradiation was significantly down-regulated after silibinin treatment both at 80 and 200 days following irradiation (Figure 5M).

### Antifibrotic effects of silibinin in irradiated lung tissue

**Figure 6:**
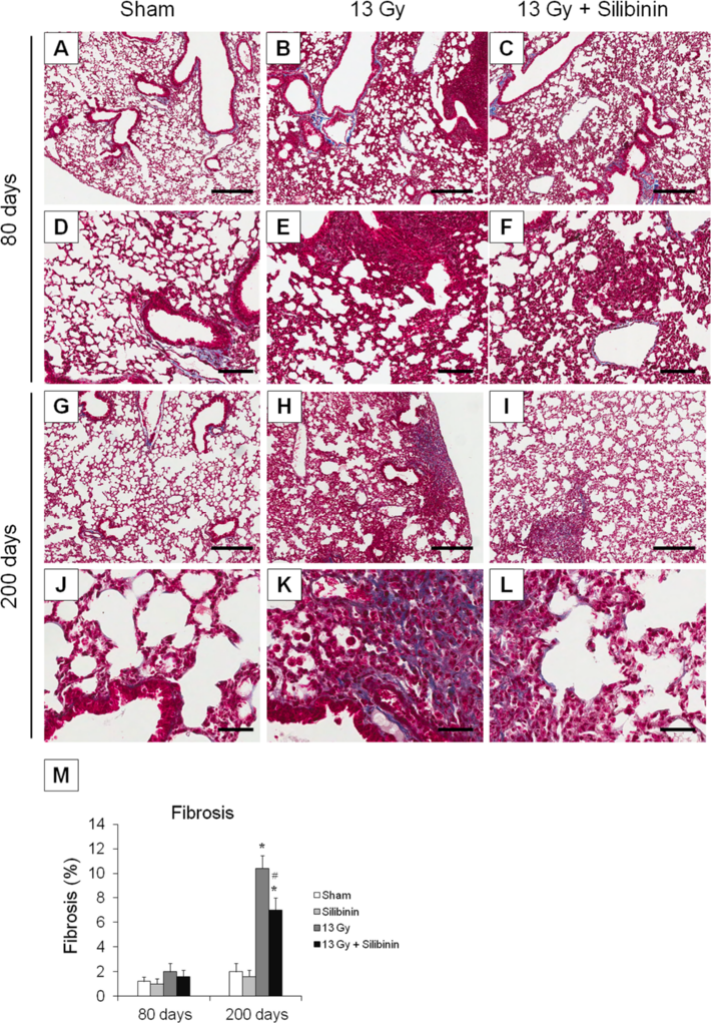
Masson’s trichrome staining for fibrosis in lung tissue sections from C57BL/6 mice treated with radiation and silibinin. **(A-F)** 80 days after irradiation; **(G-L)** 200 days after irradiation; **(A, D, G, J)** Lung tissue from sham-irradiated control; **(B, E, H, K)** Lung tissue from 13 Gy-irradiated mice; **(C, F, I, L)** Lung tissue from 13 Gy-irradiated mice with silibinin treatment. Masson’s trichome staining. Scale bars in low magnification present 300 μm. Scale bars in high magnification present 50 μm. (**M**) Percentage of fibrosis from lung tissue evaluated at 80 and 200 days following irradiation. The data are reported as means ± SEM (*n* = 5 per group). *p < 0.05 *vs.* sham-irradiated controls. ^#^ *p* < 0.05 *vs.* irradiated groups.

To determine the effect of silibinin on radiation-induced fibrosis, Masson’s trichome staining for collagen was used in the mouse lung tissue sections. In lung tissue from sham control mice, staining of collagen was only seen around vessels or bronchioles (Figure 6A and G). Collagen staining was not seen in irradiated lung tissue until 80 days after irradiation (Figure 6B), and 200 days after irradiation, extensive collagen was detected in the lung tissue of irradiated mice, indicating radiation-induced fibrosis in lung tissue (Figure 6H). In mice treated with silibinin, a significant decrease in pulmonary fibrosis was detected in lung tissue at 200 days after irradiation compared with irradiation-only group (Figure 6I and M).

### Effect of silibinin and radiation on lung cancer

To evaluate the effect of silibinin on lung cancer, LLC cells were injected into the mice through the tail vein. Mice bearing established LLC tumors were irradiated seven days after tumor injection, and silibinin was administered orally at a dose of 100 mg/day for seven days after thoracic irradiation. In the control group with untreated tumors, large tumor nodules were observed in lung tissue sections (Figure 1A). Silibinin treatment alone caused no significant differences in the number of tumor nodules, indicating that silibinin treatment alone was not related to enhancement of tumor growth (Figure 1C). In irradiated mice treated with silibinin, however, the number of tumor nodules was significantly reduced in the lungs (Figure 1C).

## Discussion

In the current study, we used a murine model to investigate the use of silibinin as a new biological strategy to reduce the adverse effects of radiotherapy for lung cancer. The results showed that silibinin has the ability to decrease inflammation and fibrosis in the lung.

Development of alveolitis and fibrosis is a known complication of thoracic radiotherapy for cancer patients. Many studies have attempted to establish the murine model of pulmonary fibrosis by irradiation [5], and have showed that radiation-induced lung response differs by genetic background. Whereas C3H strain mice with thorax irradiation only developed an inflammatory response in the lung tissue, C57BL/6 mice with thorax irradiation developed alveolitis followed by fibrosis in the late phase [4,18]. The radiation-induced pulmonary response of mice and the time to development of fibrosis in mice have been observed to be similar to human patients receiving radiotherapy [19,20]. Previous studies have shown that C57BL/6 J mice resisted death from pneumonitis after irradiation doses less than 20 Gy and died mainly of fibrosis between five and eight months. [15]. Although many agent have been investigated for ameliorating radiation effects [21,22], most studies have administered these agents prior to radiation exposure for demonstrating their efficacy as radioprotectors. However, there is a need to investigate an agent that is effective for reducing radiation-induced lung injury, because administration and radiation schedules might differ between individuals, and lung might be the target organ in possible radiological terrorism scenario with a radiolodical dispersion device [23]. In the present study, the survival rate of mice treated with 13 Gy of thorax irradiation was 50%, which was consistent with the survival rate found in previous studies [4,15,18]. However, irradiated mice treated with silibinin survived until the end of this study (200 days after irradiation), indicating the potential radioprotective effect of silibinin in normal lung tissue.

Investigation has shown that many natural compounds are able to enhance radiation-induced cancer cell destruction and protect normal tissues from radiation-induced pulmonary injury [6,24]. However, the effects of silibinin on radiation-induced lung injury remain unclear. Although the molecular and cellular mechanisms of lung injury induced by thorax irradiation are not fully determined, radiation-induced pulmonary inflammation has been suggested as a possible mechanism. Many reports have investigated radiation-induced lung damage using BALF, because BALF is thought to reflect the inflammatory response of the lung. [15,25,26]. The inflammatory response of BALF cells has been investigated in the asthmatic response and in the response to radiation [14,15]. Previous studies have suggested that inflammatory cells including neutrophils [27], lymphocytes [28], and alveolar macrophages [29] may be involved in pulmonary inflammation and fibrosis. In the present study, thorax irradiation induced a marked increase of inflammatory cells in the BALF at 80 days after irradiation and a more prominent increase at 200 days. In addition, the BALF macrophage, which is main cellular component of BALF, was shown to increase in size after irradiation; similarly, the number of multinucleated macrophages also increased after irradiation. However, silibinin treatment attenuated the increased number of inflammatory cells and morphological changes in macrophages in BALF both at 80 and 200 days after thorax irradiation, indicating that administration of silibinin suppresses the inflammatory response in the late phase after irradiation.

Thorax irradiation not only affected the inflammatory cells in the BALF, but also triggered the chronic inflammatory responses in the lung tissue. It was previously reported that pro-inflammatory cytokines, including tumor necrosis factor (TNF)-α, interleukin (IL)-1β, IL-6, and intercellular adhesion molecule 1, was induced by thorax irradiation and have an important role in development radiation-induced lung injury, including pneumonitis and fibrosis [30-33]. The mRNA levels of pro-inflammatory cytokines in the lung tissue returned to basal levels at 2 weeks, but significantly elevated at 8 weeks after irradiation with 12 Gy, indicating the biphasic expression [30]. It was also demonstrated that late cytokine expressions, such as TNF-α and IL-1β, were found at the time the mice begin to develop fibrosis in C57BL/6 mice, suggesting the involvement of inflammatory responses in the radiation-induced fibrosis [15]. In a mouse model of allergic inflammation, pretreatment of silibinin significantly attenuated the airway inflammation, via downregulation of nuclear factor-kappa B pathway [34]. In addition, silibinin has been shown to target multiple cytokine-induced signaling pathways and to down regulate iNOS expression in lung cancer [35,36]. Other studies have indicated that silibinin is pro-apoptotic, reverses cancer chemotherapy resistance and acts in combination with other agents to have a chemosensitizing effect in lung carcinoma cells [37]. In the present study, a marked injury in the lung tissue was observed and included multifocal hemorrhage and inflammatory infiltration. In addition, the presence of fibrosis was evaluated by Masson’s trichome staining, and a marked increase in fibrotic collagen tissue was shown at 200 days following thoracic irradiation. Our data showed that silibinin down-regulated the inflammation and fibrosis induced by irradiation in the lung tissue, which was correlated with the increased survival rate by thorax irradiation. The present study may provide a basis for further studies to determine the protective mechanisms of silibinin in radiation-induced lung injury, although currently we are unable to determine the precise effects of silibinin on pulmonary function. Furthermore, further studies on the role of silibinin in radiation-induced lung injury are required to determine the precise mechanisms underlying pneumonitis and fibrosis, using appropriate animal models..

A major concern of any radioprotection study is that the protective agent might theoretically protect the tumor as well, making it useless for further development. The attraction to investigate the role of silibinin as a radiosensitizer and radioprotector in lung cancer is based on its safety for clinical use. Silibinin has gained increasing attention because of its association with beneficial effects in various cancer chemoprevention [9,10,38]. Despite these studies showing the efficacy of silibinin against lung cancer, its utility in combination with radiation remains unknown. In our study, no significant difference was observed between vehicle treatment and treatment with silibinin and irradiation, indicates that silibinin does not protect tumor cells from radiation.

## Conclusions

Although cancer patients might benefit from radiotherapy, this treatment is not devoid of side effects. This study showed that in combination with radiation, silibinin increases the survival rate, possibly by reducing the inflammation and fibrosis induced by thorax irradiation, but that silibinin itself does not have a radiotherapeutic effect on tumor growth in cancer-bearing mice. Although the precise mechanism of the radioprotective effect of silibinin remains unclear, silibinin is a possible adjunct to radiation therapy and therefore might be clinically useful for lung cancer patients requiring radiotherapy.
